# Viscosity measurement dataset for a water-based drilling mud–carbon nanotube suspension at high-pressure and high-temperature

**DOI:** 10.1016/j.dib.2019.103816

**Published:** 2019-03-08

**Authors:** Kanjirakat Anoop, Reza Sadr, Rommel Yrac, Mahmood Amani

**Affiliations:** a Micro Scale Thermo Fluids (MSTF) Laboratory, Mechanical Engineering Program, Texas A&M University at Qatar, Education City, Doha, Qatar; b Department of Mechanical Engineering, Texas A&M University, College Station, TX, USA; c Petroleum Engineering Program, Texas A&M University at Qatar, Education City, Doha, Qatar

**Keywords:** Rheology, Colloidal dispersion, Drilling mud

## Abstract

This data article presents the measured viscosity of a carbon nanotube (CNT) suspension in water-based drilling mud, also termed as nano-muds (“Rheology of a colloidal suspension of carbon nanotube particles in a water-based drilling fluid” Anoop et al., 2019). The apparent viscosity values of the nano-mud samples are measured using a high-pressure high-temperature viscometer at different shear rates, working based on a rotor and bob technique. The pressure and temperature of the samples are independently varied during the measurements from ambient conditions to 171 MPa and 176 °C, respectively, within two experimental schedules. Viscosity measurements for varying nanoparticle concentration, shear rate, pressure, and temperature are reported here for different CNT concentrations.

**Table:** Specifications table

Subject area	*Mechanical and Petroleum Engineering*
More specific subject area	*Rheology*
Type of data	*Table*
How data was acquired	*Rheological data was measured using a high-pressure high temperature(HPHT) Viscometer (AMETEK Chandler engineering, Model 7600)*
Data format	*Averaged measurement data for viscosity –Analyzed data*
Experimental factors	*Viscosity measurements of water-based drilling mud samples at extreme temperatures and pressures are reported. Carbon nanotube particles are further added to these mud samples and the enhanced viscosity values are also evaluated.*
Experimental features	*Rheology of water-based drilling fluids with suspended CNT nanoparticles at high temperature and high-pressure conditions*
Data source location	*Doha, Qatar*
Data accessibility	*Data is with this article*
Related research article	K. Anoop, R. Sadr, R. Yrac, and M. Amani, "Rheology of a colloidal suspension of carbon nanotube particles in a water-based drilling fluid," *Powder Technology,* vol. 342, pp. 585–593, 2019/01/15/2019.

**Table:** 

**Value of the data** • Viscosity values of drilling mud samples with nanoparticle are measured at conditions similar to that of an actual deep-oil drilling environment. These information can be used by drilling industries in estimating the efficacy while using them in the drilling fields. • The measured viscosity values can be effectively used for theoretical development of rheological models for suspensions subjected to high-pressure and high-temperature conditions. • Nano-mud samples are colloidal dispersions and rheological measurements could be used for comparison with other colloids under high temperature and pressure conditions.

## 1 Data

Viscosity measurements from two experimental schedules [1] are presented here. Schedule I experiments corresponds to the viscosity measurements made at constant pressure (171 MPa) while varying the sample temperature. In this case, the temperature is varied from 37 °C to 176 °C. In experimental schedule II, the sample temperature is kept constant (at room temperature, 26 °C), and the pressure is varied in stages from atmospheric pressure to 171 MPa while measuring the viscosity. In both measurement schedules, data is collected at different shear rates of 5, 10, 51, 102, 170, 340, 511 and 1021 s^−1^. Measurements are recorded for each set point for a period of 1 min at a sampling rate of 1Hz. During the measurement, the maximum fluctuation in pressure and temperature is observed to be below ±0.8 MPa and ±1.2 °C respectively. Table 1, Table 2 show viscosity measurements corresponding to the *experimental schedule I* and *experimental schedule II.*

**Table 1:** Viscosity measurements from experimentation schedule I.

Experimental Schedule I							
ShearRate [s^−1^]	T [°C]	P [MPa]	Viscosity [cp]				
			Base mud	0.027 wt% NM	0.056 wt% NM	0.11 wt% NM	0.22 wt% NM
1021	37	171	4.94	19.98	20.00	25.23	35.03
511			4.41	30.50	29.88	38.32	56.53
340			4.69	41.87	41.78	51.05	75.26
170			3.40	70.44	68.65	84.24	126.53
102			2.02	112.15	110.17	129.43	185.94
51			–	208.38	205.14	240.58	333.31
1021	66	171	5.67	21.75	21.26	23.30	28.96
511			6.02	37.39	37.13	38.60	46.79
340			6.62	53.37	52.11	51.84	62.40
170			7.84	99.52	95.56	90.37	105.79
102			8.00	170.25	160.80	147.91	164.21
51			9.00	334.85	320.20	287.65	296.41
1021	93	171	6.39	28.21	25.98	25.01	25.76
511			7.42	50.49	49.30	44.79	43.49
340			9.41	75.27	73.60	65.54	58.89
170			14.08	148.26	142.38	123.43	102.18
102			19.98	246.41	237.16	203.69	160.08
51			35.44	494.53	474.43	406.11	296.98
1021	121	171	6.82	28.44	25.60	25.15	23.37
511			8.31	56.40	51.81	49.28	42.29
340			10.94	83.67	78.73	74.63	59.76
170			16.90	167.46	154.47	147.90	111.27
102			25.26	283.70	261.39	245.45	185.45
51			44.91	581.64	531.25	480.87	341.73
1021	150	171	5.53	23.19	20.44	21.94	20.91
511			6.74	44.74	38.57	40.95	39.18
340			9.11	63.52	55.03	58.33	53.77
170			15.94	116.30	100.09	106.20	101.40
102			22.01	183.34	153.24	163.40	172.16
51			42.18	350.88	285.11	308.66	344.09
1021	176	171	2.91	16.49	15.23	17.05	19.23
511			5.01	25.94	25.82	27.42	35.44
340			7.10	35.93	34.42	39.33	51.09
170			13.17	69.55	63.66	76.65	108.29
102			21.57	110.81	98.78	121.47	173.69
51			43.07	210.67	182.79	231.58	294.55

**Table 2:** Viscosity measurements from experimentation schedule II.

Experimental Schedule II							
Shear Rate [s^−1^]	T [°C]	P [MPa]	Viscosity [cp]				
			Base mud	0.027 wt% NM	0.056 wt% NM	0.11 wt% NM	0.22 wt% NM
1021	26	0	5.52	13.32	15.76	16.25	24.70
511			6.05	18.70	21.83	19.15	29.93
340			7.43	25.52	28.53	22.66	35.06
170			9.76	34.89	43.76	31.04	48.43
102			10.66	55.78	64.74	40.76	62.98
51			13.72	86.67	104.92	55.18	88.80
1021	26	33	6.52	13.91	15.66	16.13	22.00
511			8.04	18.51	21.31	20.10	28.00
340			10.11	25.65	27.63	24.95	34.12
170			16.71	35.08	42.12	36.47	50.12
102			25.27	53.69	63.71	52.50	67.67
51			44.49	93.56	104.99	75.15	97.56
1021	26	68	6.81	13.97	16.40	16.39	21.02
511			8.17	18.70	21.57	20.60	27.42
340			10.22	26.34	28.24	26.08	34.06
170			16.30	38.40	43.37	38.47	50.96
102			25.46	58.57	66.72	55.80	70.14
51			38.21	95.07	108.18	87.60	106.81
1021	26	103	7.13	13.86	17.03	16.67	20.13
511			8.44	18.24	22.65	21.29	27.60
340			10.63	23.86	29.64	27.40	35.07
170			16.68	36.19	46.20	41.35	53.09
102			24.04	50.05	69.46	61.64	73.27
51			37.33	84.65	116.05	101.40	113.43
1021	26	138	7.62	14.41	18.00	17.71	20.08
511			9.35	19.39	24.64	23.34	27.75
340			12.20	25.66	33.10	30.32	35.96
170			19.55	39.25	51.49	45.67	55.03
102			28.28	58.46	78.11	66.01	76.15
51			50.22	103.06	136.99	109.16	121.74
1021	26	171	8.27	15.31	19.68	18.51	21.29
511			10.16	21.13	27.57	24.76	30.08
340			13.15	28.62	37.63	33.22	39.59
170			20.90	45.24	58.99	50.86	61.21
102			27.06	68.44	88.93	74.69	83.61
51			38.68	120.59	153.59	130.61	133.34

## 2 Experimental design, materials and methods

Viscosity measurements of a water-based drilling mud sample and colloidal suspensions formulated by dispersing carbon nanotube particles (CNT) in them are reported here. The nanoparticle-mud mixture (termed as CNT-nano-muds) is prepared by ultrasonication of commercially purchased nanoparticles into the drilling mud. For this, first, the water-based drilling mud (termed as the *basemud*) is prepared. The basemud contains 1% of barite and 7% of bentonite by weight and the mud density is 12.5 ppg (pounds per gallon). The bentonite is initially mixed with the drill water and is aged for approximately 16 hours of hydration to form pre-hydrated bentonite (PHB). The weighing agent (barite) is incorporated in the last 10 minutes of mixing. The CNT-nanomud samples are then prepared by adding appropriate amounts of carbon nanotubes into the basemud. Commercially procured (773840 Aldrich) multi-walled carbon nanotubes (≥98% carbon basis, O.D × I.D × L- 10nm± 1 nm × 4.5 nm ± 0.5nm × 3–6μm) are used to prepare the nanomuds samples. A stable dispersion is achieved by using an intensified ultrasonication with a probe-type sonicator (QSonica S-4000, 20 kHz) to de-agglomerate particles in the solution. The nanomud suspension was sonicated for 40 minutes. To control the temperature increase during the sonication process, the sample was kept in a temperature controlled bath maintained at 15 °C. Apart from the basemud, viscosity measurements of four CNT-nanomud sample concentration of 0.027%, 0.056%, 0.11% and 0.22% by weight (termed as *wt% NM*) are presented here.

The state-of-the-art high-pressure high-temperature (HPHT) viscometer (Model 7600, AMETEK Chandler engineering) is used for measuring the rheological characteristics of the nano-mud samples. The high pressure and high-temperature environment similar to that occurring at oil well conditions are created during the measurement with considerable safety precautions together with satisfying the ISO and API standards. This couette type viscometer is fully automated and uses a rotor and bob (1.725 cm radius and 3.805 cm height) geometry for the measurement of the apparent viscosity of the sample. The fluid sample fills in the annular space between the bob and rotor cylinders. A precision torsion spring and a high-resolution encoder measure the shear stress created between a stationary bob and the rotating rotor. Stipulated shear rates are established between the bob and rotor using predefined bob/rotor geometry with rotational speeds ranging from 0 to 900 rpm (0–1533 sec^−1^). The outer side of the rotor has a helical screw design which helps in the mixing of the sample during the entire experimental schedule. The apparent viscosity is estimated as the ratio of shear stress to shear rate. Further information on the viscometer and its construction could be seen in our earlier works [2], [3].
