# Supplementary material for: Viscosity measurement dataset for a water-based drilling mud–carbon nanotube suspension at high-pressure and high-temperature
Source: Data Brief. 2019 Mar 8;24:103816. doi: 10.1016/j.dib.2019.103816 (PMC6463761; doi:10.1016/j.dib.2019.103816)
Supplement: Multimedia component 1 [file mmc1.pdf]

This is to declare that the authors of the manuscript "Viscosity measurement dataset for a water-based drilling mud–carbon nanotube suspension at high-pressure and high-temperature" have no competing interest to declare.
